# Phytosteryl Esters Encapsulated in Liposomes as Bioactive Additives in Mayonnaise

**DOI:** 10.3390/molecules31152687

**Published:** 2026-08-02

**Authors:** Magdalena Rudzińska, Joanna Igielska-Kalwat, Anna Grygier, Aleksander Siger, Anna Misiak

**Affiliations:** Faculty of Food Science and Nutrition, Poznań University of Life Sciences, 60-624 Poznan, Poland; joanna.igielska-kalwat@up.poznan.pl (J.I.-K.); anna.grygier@up.poznan.pl (A.G.); aleksander.siger@up.poznan.pl (A.S.); 90868@student.up.poznan.pl (A.M.)

**Keywords:** phytosterol, liposome, mayonnaise, heating, storage, thermo-oxidative stability

## Abstract

**Background**: Phytosterols are natural compounds that provide health benefits by lowering LDL cholesterol levels, but they are susceptible to thermo-oxidative degradation during food storage and processing. Encapsulating them in liposomes could increase their stability and bioavailability. The aim of this study was to examine the stability of phytosterols encapsulated in liposomes during heating of mayonnaise at temperatures simulating storage and frying conditions. **Methods**: Mayonnaise containing 2, 4, 6, and 8% liposomes encapsulating stigmasterol oleate was prepared and heated at 60 °C for 8 h and at 180 °C for 30, 60, 90, and 120 min. The fat content, percentage composition of fatty acids and levels of oleic acid and stigmasterol were determined in each tested mayonnaise. **Results**: The fat content of mayonnaise with liposomes heated at 60 °C for 8 h was 97–98%. During heating at 180 °C for 60 min, it reached 100%, and after 120 min, it decreased to 75%. The oleic acid content of liposome-enriched mayonnaise remained stable during heating. The greatest loss of stigmasterol was observed in the control sample without liposomes, in which the sterol content decreased 79%. After heating mayonnaise with liposomes, the decrease in stigmasterol ranged from 3 to 25% at 60 °C for 8 h and from 90% to 100% at 180 °C for 90 and 120 min. **Conclusions**: Enriching mayonnaise with phytosterol esters encapsulated in liposomes can effectively improve the thermal-oxidative stability of plant sterols during short-term heating, but does not protect the product from the loss of these sterols during prolonged, intense heating.

## 1. Introduction

**Figure 1:**
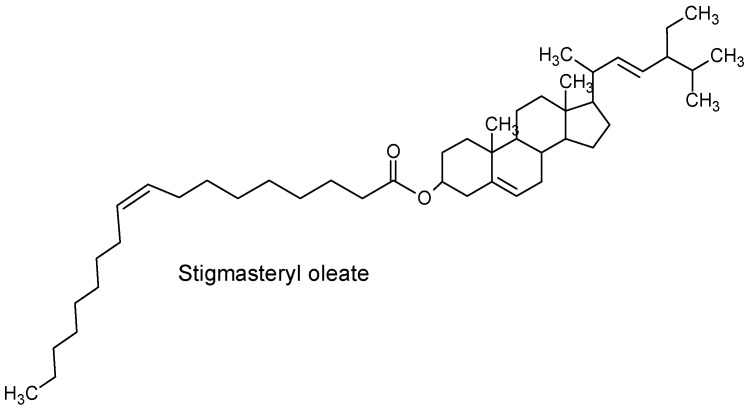
Chemical structure of stigmasteryl oleate.

Mayonnaise is a type of oil-in-water emulsion prepared mainly from vegetable oils, egg yolk, vinegar, citric or acetic acid, and some permitted additives. Edible vegetable oil accounts for about 70% of mayonnaise’s composition and is a source of energy, unsaturated fatty acids and phytosterols. Because of egg yolk addition, cholesterol is also one of the most important components of mayonnaise. The dietary cholesterol was included in the United States Dietary Guidelines (USDG) Technical Report’s list of “nutrients of concern” in relation to the chronic disease burden [1]. Wide clinical and epidemiological evidence supports a direct link between high plasma cholesterol levels, particularly low-density lipoprotein (LDL) cholesterol, and cardiovascular disease (CVD) risk [2]. Phytosterols from vegetable oils inhibit the absorption of cholesterol from egg yolk, but intestinal absorption of plant sterols is much lower than that of cholesterol. Phytosterols and their saturated forms, named as phytostanols, are structurally similar to cholesterol, although they differ in the complexity of the side chain attached to the ring D (Figure 1). Unfortunately, phytosterols are poorly absorbed in the intestine (0.4–3.5%), while phytostanols are absorbed only 0.02–0.30% [3]. In contrast, the intestinal absorption of cholesterol ranges from 35 to 70% [4]. The cholesterol-lowering properties of phytosterols and phytostanols have been demonstrated in humans and animals, and their consumption is recommended by the National Cholesterol Education Program [5] as a therapeutic dietary option to lower LDL cholesterol. Phytosterol esters reduce the absorption of dietary and biliary cholesterol. Consuming 2–3 g/day of plant sterols and stanols can lower LDL cholesterol levels by 9–12% [6]. According to the ESC/EAS guidelines, foods fortified with phytosterols at a dose of 1–2 g/day may support the treatment of hypercholesterolemia in patients at low cardiovascular risk [7].

The use of phytosterols for food fortification requires effective extraction methods that ensure high purity and stability. The main source of phytosterols for the food industry is deodorization distillate (DOD), a byproduct of oil refining. Traditional methods for extracting phytosterols include solvent extraction, often followed by saponification and esterification to improve their solubility in fats [8]. Phytosterols are susceptible to oxidation during thermal processing and storage, yielding potentially harmful derivatives with health risks, including cytotoxicity and pro-inflammatory effects [9].

Thermo-oxidative degradation of phytosteryl esters is substantially affected by the degree of fatty acid unsaturation and by typical reaction parameters, such as temperature and time. The unsaturation of fatty acids affected the degradation rate of stigmasteryl esters. The kinetics of stigmasteryl stearate and oleate degradation were similar, whereas the additional double bonds in linoleic and linolenic esters of stigmasterol resulted in faster decomposition. The sterol and fatty acid molecules degraded at different rates, with the fatty acid moiety degrading faster than the sterol at both temperatures, independent of heating time and degree of unsaturation [10].

Liposomes encapsulated with free phytosterols used for non-heated foods were better than esters, but food products subjected to frying were the least affected when oleic acid esters of phytosterols were used [11]. Gastrointestinal digestion increased the cytotoxicity of liposomes with free stigmasterol, but liposomes with stigmasterol esters had lower cytotoxicity than undigested liposomes [12].

The mayonnaise market is currently experiencing rapid growth, driven by shifting consumer preferences and growing interest in health-conscious products. At the same time, it is characterized by a growing preference for convenient, ready-to-use products. Manufacturers are responding to this trend with recipe innovations and mayonnaise is increasingly serving not only as a sandwich spread but also as an ingredient in sauces, dips, salads and convenience foods. Enriching mayonnaise with liposome-encapsulated phytosterols may help enhance its health-promoting properties [13]. By increasing the bioavailability of phytosterols, we can reduce the amount that needs to be consumed while achieving a similar cholesterol-lowering effect. Encapsulating phytosterols in liposomes will not only increase the sterols’ oxidative stability but also their bioavailability.

The aim of this study was to examine the stability of stigmasteryl oleate (Figure 1) encapsulated in liposomes during the heating of mayonnaise at temperatures simulating storage and frying conditions.

## 2. Results

### 2.1. Fat Content

**Figure 2:**
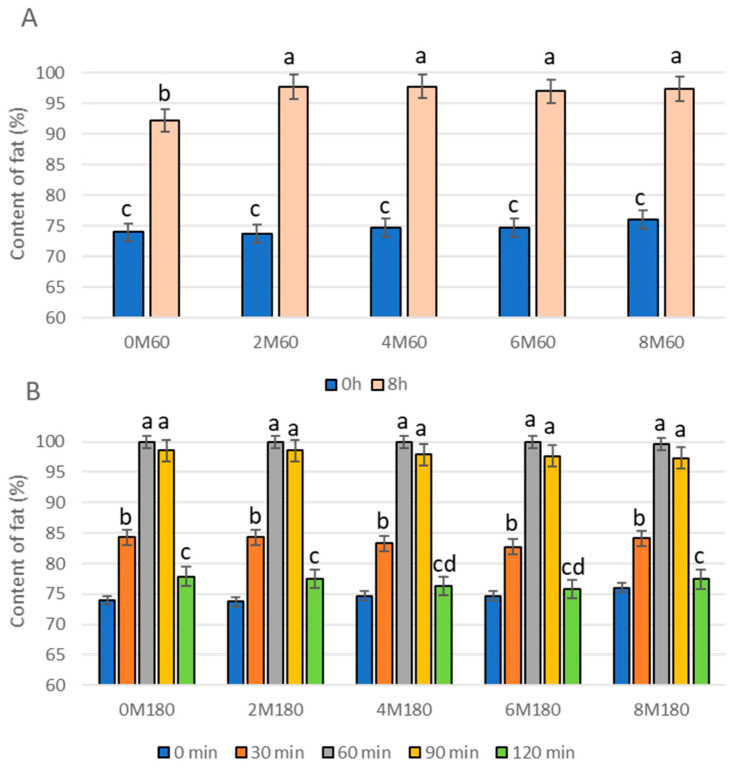
Fat content in mayonnaise samples enriched in 2, 4, 6, and 8% of liposomes encapsulated with stigmasterol oleate after heating at 60 °C for 8 h (**A**) and at 180 °C for 30, 60, 90, and 120 min (**B**). 0M—mayonnaise without liposomes; 2M—mayonnaise with 2% of liposomes; 4M—mayonnaise with 4% of liposomes; 6M—mayonnaise with 6% of liposomes; 8M—mayonnaise with 8% of liposomes. Bars marked with different superscript letters indicate statistically significant differences among treatments (*p* < 0.05; one-way ANOVA followed by Tukey’s post hoc test).

Prepared samples of mayonnaise containing liposomes encapsulating stigmasterol oleate were heated at 60 °C for 8 h and 180 °C for 30, 60, 90, and 120 min. The obtained results are presented in Figure 2. After 8 h of heating at 60 °C, all liposome-containing samples showed a significantly higher fat content than the control sample (0%). The fat content in samples containing 2–8% liposomes remained at a constant, high level (~97–98%), whereas in the control sample it was lower at 92%. During heating at 180 °C for 30 and 60 min, the fat content increased steadily, reaching 83–84% after 30 min and 100% after 60 min. Subsequently, the fat content decreased to 97–98% after 90 min and to 75–77% after 120 min. No significant differences were observed between the control sample and the mayonnaise samples with added liposomes. The measured fat content after heat treatment may have been partially overestimated due to water evaporation from the mayonnaise, which increased the concentration of dry-matter components. Consequently, the observed differences should be interpreted as changes in the water and fat content of the tested system, rather than solely as an absolute change in lipid content.

Phytosterols, as well as TAG and other lipid components, degrade during heating mainly through oxidation, hydrolysis, and thermal processes (primarily polymerization and cyclization). One of the first reactions observed during thermal processes is oxidation. The oxidation products form compounds with different molecular weights. The high-molecular-weight compounds, like dimers and oligomers, are formed, and they can be difficult to dissolve. The decrease in fat content in mayonnaise heated at 180 °C for 90 and 120 min is likely due to the polymerization of lipid components.

### 2.2. Fatty Acids

**Figure 3:**
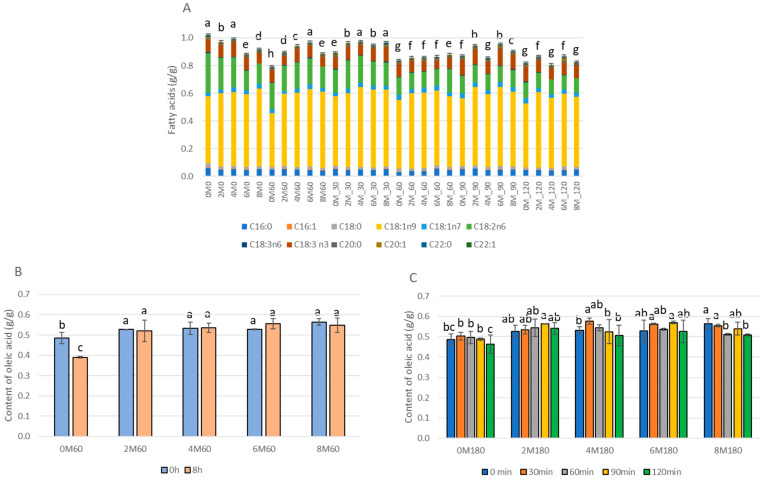
(**A**)—The percentage composition and the total content of fatty acids in lipids extracted from mayonnaise samples; (**B**)—the content of oleic acid (C18:1n9) before and after heating of each mayonnaise at 60 °C for 8 h, and (**C**)—at 180 °C for 30, 60, 90, and 120 min. 0M—mayonnaise without liposomes; 2M—mayonnaise with 2% of liposomes; 4M—mayonnaise with 4% of liposomes; 6M—mayonnaise with 6% of liposomes; 8M—mayonnaise with 8% of liposomes. Bars marked with different superscript letters indicate statistically significant differences among treatments (*p* < 0.05; one-way ANOVA followed by Tukey’s post hoc test).

Twelve fatty acids were identified in the fat extracted from the mayonnaise samples (Figure 3A) and their percentage composition was typical of rapeseed oil. Oleic acid (C18:1n9) was the predominant fatty acid in the tested mayonnaise, accounting for 49% of the total fatty acid content in the control sample (0M0). Linoleic acid accounted for 28%, linolenic acid for 9%, and palmitic acid for 7%. The addition of liposomes did not significantly affect the fatty acid profile of the tested mayonnaise. Heating at 60 °C for 8 h and at 180 °C for 30–120 min did not cause significant changes in the fatty acid composition. The use of liposomes containing stigmasterol oleate did not significantly alter the product’s lipid structure and their presence may have helped maintain the stability of the mayonnaise under heat-treatment conditions.

The determined oleic acid originated from both the fat used and the added liposomes. Therefore, it was decided to thoroughly analyze the thermal and oxidative changes in this acid in the mayonnaise under study. Based on the data obtained, it was found that in unheated mayonnaise samples, the C18:1n9 content increased with an increase in the proportion of liposomes, from the value observed in the control sample without the additive (0.46 g/g) to the highest levels in the mayonnaise containing 8% liposomes (0.56 g/g). This indicates that the addition of liposomes containing stigmasterol oleate contributed to an increase in the proportion of the monounsaturated fraction in the total pool of fatty acids.

Heating mayonnaise without liposomes (0M0) at 60 °C for 8 h resulted in a decrease in the oleic acid content of the liposome-free mayonnaise (0M60), suggesting that C18:1n9 is susceptible to degradation or transformation under the influence of prolonged, moderate heating. In mayonnaise samples containing liposomes (2–8M60) heated at 60 °C, the C18:1n9 content was higher than in the 0M60 sample and similar to the values observed in the corresponding unheated samples, which may indicate a stabilizing effect of liposomes on oleic acid under these conditions (Figure 3B). In samples heated at 180 °C for 30–120 min, the C18:1n9 content remained at a similar level, with no significant changes as treatment time increased (Figure 3C). In both the control samples and those containing 2–8% liposomes, the C18:1n9 level remained at a similar level. The observed differences did not indicate significant degradation of this fatty acid during the study period. In several variants with added liposomes (particularly at 4–8% concentration), slightly higher C18:1n9 values were recorded compared to samples without liposomes, suggesting that liposomes may, to a limited extent, protect oleic acid from adverse changes during intensive heat treatment. Based on the results obtained, it can be concluded that the addition of liposomes containing stigmasterol oleate not only did not reduce the C18:1n9 content in mayonnaise but, in many cases, helped maintain or moderately increase it, under both mild and high-temperature heating conditions. This effect helps preserve the monounsaturated fatty acid profile in a product subjected to heat treatment. Microencapsulation is widely used to protect lipid compounds; however, among the many techniques, spray drying is the most commonly used. The microencapsulation of fish oil, chia oil, and walnut oil required spray drying, which involved heating the liposomes to 170–180 °C for a short period of time [14,15,16]. Studies have demonstrated the stability of polyunsaturated fatty acids (PUFAs) during this process; however, no research has examined their stability as food ingredients during storage and processing.

### 2.3. Sterol

The stigmasterol content of the rapeseed oil used was 6.5 mg/g, whereas in the mayonnaise immediately after preparation, it was 5.9 mg/g. The fat extracted from the mayonnaise contained small amounts of cholesterol, which confirmed the presence of a fat fraction derived from egg yolk.

**Figure 4:**
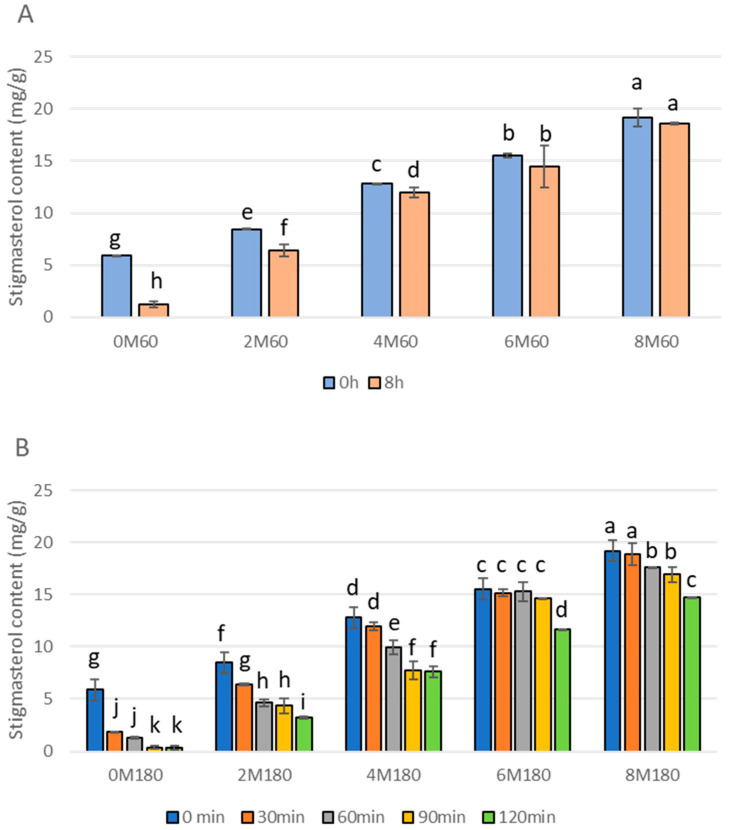
Content of stigmasterol in mayonnaise samples enriched in 0, 2, 4, 6, and 8% of liposomes before and after heating at 60 °C for 8 h (**A**), and at 180 °C for 30, 60, 90, and 120 min (**B**). 0M—mayonnaise without liposomes; 2M—mayonnaise with 2% of liposomes; 4M—mayonnaise with 4% of liposomes; 6M—mayonnaise with 6% of liposomes; 8M—mayonnaise with 8% of liposomes. Bars marked with different superscript letters indicate statistically significant differences among treatments (*p* < 0.05; one-way ANOVA followed by Tukey’s post hoc test).

The addition of stigmasterol-containing liposomes significantly increased the stigmasterol level in the product. The use of 2% liposomes raised the stigmasterol content to 8.5 mg/g; at 4%, it was 12.8 mg/g; at 6%, 15.5 mg/g; and at 8%, 19.2 mg/g (Figure 4). When the mayonnaise was heated at 60 °C for 8 h, the greatest loss of stigmasterol was observed in the control sample without liposomes, in which the sterol content decreased 79% (Figure 4A). In samples with added liposomes, the decrease was significantly smaller, ranging from 3 to 25%. The greatest decrease in stigmasterol levels in the sample without liposomes indicates the instability of this compound during storage and heat treatment of mayonnaise, likely due to degradation of the sterol present in rapeseed oil. Stigmasterol, added as liposomes, exhibited significantly greater thermal and oxidative stability and may also have limited the degradation of other fat components in mayonnaise.

During heating at 180 °C, the loss of stigmasterol in mayonnaise without added liposomes was very rapid: after 30 min, its content decreased by 68%; after 60 min, by 78%; and after 90 and 120 min, it exceeded 90% (Figure 4B). In samples containing 2% liposomes, stigmasterol was significantly more stable: after 30 min of heating, the loss was 25%; after 120 min, 62%. With 4% liposomes, a further improvement in stability was observed: after 30 and 60 min of heating, the loss was 7% and 22%, respectively, while extending the heating time from 90 to 120 min no longer significantly affected the stigmasterol level (a 40% loss at both time points).

The smallest decrease in stigmasterol content was observed in mayonnaise with a 6% liposome addition. In this sample, the sterol concentration remained stable during the first 90 min of heating and a loss did not occur until 120 min, when it amounted to 25%. In mayonnaise with an 8% liposome addition, stigmasterol degradation was the lowest among all samples; however, after 120 min of heating, the loss was 23%, a value similar to that of the sample with 6% liposomes.

Mayonnaise containing phytosterol ester taken for 3 months lowered serum cholesterol more than conventional mayonnaise [17]. The addition of phytosterol esters encapsulated in liposomes may also significantly lower cholesterol levels; however, their use in food requires investigation of the matrix’s effect on their stability.

Degradation of lipids during storage and heating occurs mainly through oxidation, hydrolysis, and thermal processes (primarily polymerization and cyclization). The oxidative stability of mayonnaise, including that of formulations with added phytosterols, has been demonstrated and described by many authors [18,19,20].

Degradation of phytosteryl esters encapsulated in liposomes and formation of derivatives were described [11]. Liposomes encapsulated with free stigmasterol and its esters with myristic and oleic acids were heated at 60 and 180 °C. Stigmasterol oxidation derivatives, dimers and oligomers were determined. The results show that using liposomes containing free phytosterols in non-heat-treated foods is preferable to using esters. Food products subjected to frying, on the other hand, will be least likely to be adversely affected when phytosterol esters with oleic acid are used.

The cytotoxicity of liposomes encapsulated with stigmasteryl oleate was described by Rudzińska et al. [12]. The presented results demonstrated that the cytotoxicity of liposomes can be ranked in the following order: liposomes encapsulated with free stigmasterol < liposomes encapsulated with stigmasteryl oleate < liposomes encapsulated with stigmasteryl myristate.

## 3. Materials and Methods

### 3.1. Materials

Stigmasterol (≥95%), oleic acid (≥99%), all solvents, sodium hydroxide, anhydrous pyridine, the catalysts dicyclohexylcarbodiimide (DCC) and 4-dimethylaminopyridine (DMAP), high-purity silica gel 70–230 mesh, standards of cholesteryl oleate (98%), 5α-cholestane, and heptadecanoic acid methyl ester were all purchased from Sigma-Aldrich (St. Louis, MO, USA). The silylation mixture of BSTFA [*N*,*O*-Bis(trimethylsilyl) trifluoroacetamide] with 1% TMCS (trimethylchlorosilane) was obtained from Fluka Chemie AG (Buchs, Switzerland), while the SEP-PAK amino cartridges were sourced from Waters (Milford, USA). Dipalmitoylphosphatidylcholine (DPPC) was purchased from Avanti Polar Lipids (Birmingham, AL, USA). Refined rapeseed oil (‘Kujawski’ brand) and eggs were purchased from a local retail market.

### 3.2. Esterification

Our previous studies have shown that esters of oleic acid and phytosterols are the most stable during food storage and processing [11,12]. In this study, liposomes containing stigmasterol oleate were used. Chemical esterification was used to obtain the stigmasteryl oleate [10,21]. Briefly, stigmasterol was dissolved in dichloromethane and the catalysts (500 mg DCC and 15 mg DMAP) and oleic acid were added. Esterification was run in argon at room temperature for 24 h in the dark. Distilled water was added to the finished reaction and the mixture was separated in a separatory funnel. The lower layer was collected and the cleaning procedure was repeated three times. The solvent was evaporated and the residue was dissolved in hexane. The resulting mixture was purified on a silica gel column and purity was verified by TLC, using cholesteryl oleate as a reference. The quality of stigmasteryl oleate was determined using 1H-NMR and GC–MS.

### 3.3. Preparation of Liposomes

A chloroform:methanol solution of DPPC and stigmasterol oleate was dried under a stream of nitrogen [22]. The dry lipid films were hydrated by adding 1 mL of buffer at a temperature 10 °C above the gel–liquid–crystalline phase-transition temperature of the prepared liposomes. The final lipid concentration in phosphate buffer ranged from 10 to 30 mg/mL. The liposomal suspensions were treated 10 times with a heating/cooling process: the liposomes were incubated for 10 min at 4 °C, then heated to a temperature 10 °C above the Tm of the doped liposomes and incubated at this temperature for 10 min. The liposome morphology was standardized using sonication. The characterization, stability and formation of derivatives were described [11].

### 3.4. Freeze-Drying of Liposomes

The liposomes were dried using an Alpha 2–4 LD freeze-dryer (Christ, Germany), freezing at −18 °C and drying at −20 °C for 20 h. Desiccation was then performed at 5 °C for 4 h [23].

### 3.5. Mayonnaise Preparation

The egg yolk (12 g), vinegar (8 g), salt (3.6 g), and sugar (10 g) were placed in the homogenizer vessel. 300 mL of oil and 62.4 mL of water were added, and the mixture was homogenized. The mayonnaise was then divided into five parts, and freeze-dried liposomes were added at 2%, 4%, 6%, and 8% to each part. Mayonnaise without added liposomes was used as the control sample. The experiment was performed in two replicates. Fat, fatty acid, and sterol content were analyzed in triplicate for each of the two independent batches, yielding six measurements per condition.

### 3.6. Storage Stability

Mayonnaise samples containing liposomes encapsulated with stigmasteryl oleate were heated at 60 °C to simulate storage conditions, in line with AOCS Official Method Cg 5-97 [24]. Samples were placed into glass ampules and heated to 60 °C for 8 h. After heating, the ampoules were sealed, cooled to room temperature and stored in the fridge for up to 24 h prior to analysis. The experiment was performed in two replicates. Fat, fatty acid, and sterol content were analyzed in triplicate for each of the two independent batches, yielding six measurements per condition.

### 3.7. Thermal Stability

Mayonnaise samples were heated to 180 °C to determine their thermal stability under simulated frying conditions. Briefly, the samples were placed into glass ampules and heated at 180 °C for 30, 60, 90, and 120 min. After heating, the ampoules were sealed, cooled to room temperature and placed in the fridge. The samples were stored for analysis, though not for longer than 24 h. The experiment was performed in two replicates. Fat, fatty acid, and sterol content were analyzed in triplicate for each of the two independent batches, yielding six measurements per condition.

### 3.8. Fat Content

Fat content in the examined mayonnaise samples was determined using the Grossfeld method. A 2 g sample of homogenized material was quantitatively transferred to a 250 cm^3^ conical flask, followed by the addition of 15 cm^3^ of concentrated hydrochloric acid. The flask was fitted with a reflux condenser and placed in a boiling water bath for 15 min, with the contents mixed every 5 min. After hydrolysis, the sample was cooled under running water, 10 cm^3^ of ethanol was added, followed by 50 cm^3^ of petroleum ether. The flask was sealed, gently shaken to avoid emulsion formation and left until a clear separation of the aqueous and ether layers was achieved. The ether extract was pipetted into a previously dried and weighed weighing dish. The solvent was evaporated on a water bath, and the dish was placed in a laboratory oven at 105 °C for 1 h. After cooling in a desiccator, the fat residue was weighed on an analytical balance. Fat content was calculated from the mass of the residue after evaporation, then recalculated relative to the total volume of solvent used and the sample weight, assuming a fat density of d = 0.92 g/cm3, which is appropriate for the vegetable fats used in mayonnaise production. It should be noted that the Grossfeld method quantifies total extractable lipids and does not distinguish triacylglycerols from added DPPC phospholipids; this should be considered when interpreting fat content values in liposome-enriched samples.

### 3.9. Sterols

Sterol content was determined using AOCS Official Method Ch 6-91 [25]. Liposomes were dissolved in chloroform to release the encapsulated substances. The solvent was then evaporated under nitrogen steam and 1 mg of encapsulated substances was saponified. The unsaponifiable fraction was extracted using hexane:methyl *tert*-butyl ether (1:1, *v*/*v*). After evaporation, the solvent samples were silylated, and the sterols were determined on a gas chromatograph HP 6890 equipped with a DB-35MS capillary column (25 m × 0.20 mm × 0.33 µm; J&W Scientific, Folsom, CA, USA). Samples were injected at 290 °C in splitless mode. The column temperature was programmed from 100 °C, held for five minutes, then increased at 25 °C/min to 250 °C, held for one minute, and then increased at 3 °C/min to 290 °C, held for twenty minutes. The detector temperature was set to 300 °C. Hydrogen was used as a carrier gas at a flow rate of 1.5 mL/min. 5α-Cholestane was used as an internal standard. Stigmasterol was quantified with a general relative response factor of 1.00, which was confirmed using commercially available stigmasterol standards. Stigmasterol was identified and quantified by comparison with the standard’s retention data.

### 3.10. Fatty Acids

Fatty acid content was determined using the AOCS Official Method Ce 1 k-07 [26]. Liposomes were dissolved in chloroform to release the encapsulated substances, and the solvent was then evaporated under a nitrogen stream. The residue was hydrolyzed with 0.5 M KOH in methanol and then methylated with 14% boron trifluoride as the catalyst. For separation, an Agilent 8890 gas chromatograph (Agilent Technologies, Santa Clara, CA, USA) equipped with a HP-88 capillary column (100 m × 0.25 mm × 0.20 μm; Agilent Technologies, Santa Clara, CA, USA). Samples were run in split mode (50:1), and the injector and detector were held at 250 °C. The column temperature was initially held at 160 °C for 1 min. The temperature was then increased at 6 °C/min to 220 °C and held for 17 min. Hydrogen was used as the carrier gas at a flow rate of 1.0 mL/min. Fatty acids were quantified using methyl heptadecanoate as the internal standard. Retention data for fatty acid methyl ester standards were compared to identify them.

### 3.11. Statistical Analysis

The experiments and analyses were performed in two independent replicates and the data presented are mean values with standard deviation (±SD). Statistical analysis was performed using the STATISTICA version 13.3 software (Statsoft, Inc., Tulsa, OK, USA). The significance of the main effects was determined by One-way analysis of variance (ANOVA). Fat, fatty acid, and sterol content were analyzed in triplicate for each of the two independent batches, yielding six measurements per condition.

## 4. Conclusions

The obtained results clearly indicate that encapsulating stigmasterol in liposomes significantly improves its stability in a mayonnaise-type emulsion, both under storage conditions and during moderate heat treatment. Even a small addition of liposomes (2%) reduced stigmasterol losses compared to the control sample. Further increasing the liposome content (4–8%) resulted in a gradual decrease in the rate of sterol degradation.

The observed protective effect can be attributed to several mechanisms: the physical separation of stigmasterol molecules from the oil phase, which is most vulnerable to oxidation, the restriction of oxygen access to the sterol enclosed within the liposome structure, and the potential interaction of liposome membrane components (phospholipids) on local redox conditions in the emulsion system.

It is important to note, however, that under conditions of prolonged heating, especially at elevated temperatures, the degradation of stigmasterol cannot be avoided, even at high liposome concentrations. This suggests a limit to protective efficacy, resulting from the exhaustion of the liposomes’ structural capacity and the intensification of degradation processes within the fat system. Enriched mayonnaise with phytosterols in liposomal form can effectively improve the stability of encapsulated compounds during short-term heating (e.g., heating the sauce or brief exposure to elevated temperatures), but does not protect the product from sterol losses during prolonged, intense heating.

The use of liposomes as phytosterol carriers appears to be a promising direction in mayonnaise and emulsion sauce technology; however, further research should focus on evaluating the impact of various process parameters (pH, aqueous phase composition, type of oil) and comparing liposomes with other carrier systems (e.g., nanoemulsions, micro- and nanoencapsulation in carbohydrates or proteins). Only such a comprehensive analysis will allow the full exploitation of liposomes’ potential to protect phytosterols, while ensuring high sensory quality and stability of the final product.

## Data Availability

https://repod.icm.edu.pl/dataset.xhtml?persistentId=doi:10.18150/G2QMTV (accessed on 29 June 2026).

## Abbreviations

The following abbreviations are used in this manuscript:

0M60	Mayonnaise without the addition of liposomes, heated at 60 °C
2M60	Mayonnaise with 2% of liposomes, heated at 60 °C
4M60	Mayonnaise with 4% of liposomes, heated at 60 °C
6M60	Mayonnaise with 6% of liposomes, heated at 60 °C
8M60	Mayonnaise with 8% of liposomes, heated at 60 °C
0M180	Mayonnaise without the addition of liposomes, heated at 180 °C
2M180	Mayonnaise with 2% of liposomes, heated at 180 °C
4M180	Mayonnaise with 4% of liposomes, heated at 180 °C
6M180	Mayonnaise with 6% of liposomes, heated at 180 °C
8M180	Mayonnaise with 8% of liposomes, heated at 180 °C
